# Recycling at synapses

**DOI:** 10.7554/eLife.17692

**Published:** 2016-06-29

**Authors:** Owen P Gross, Henrique von Gersdorff

**Affiliations:** The Vollum Institute, Oregon Health and Science University, Portland, United States; The Vollum Institute, Oregon Health and Science University, Portland, United Statesvongersd@ohsu.edu

**Keywords:** exocytosis, endocytosis, synaptic terminals, image analysis, electrophysiology, transgenic animals, Mouse, Rat

## Abstract

Synaptic vesicles in rodent neurons are recycled using at least two distinct mechanisms.

**Related research article** Okamoto Y, Lipstein N, Hua Y, Lin KH, Brose N, Sakaba T, Midorikawa M. 2016. Distinct modes of endocytotic presynaptic membrane and protein uptake at the calyx of Held terminal of rats and mice. *eLife* **5**:e14643. doi: 10.7554/eLife.14643

**Image** The slower mode of vesicle recycling can take up to tens of seconds, whereas a faster mode takes a few seconds

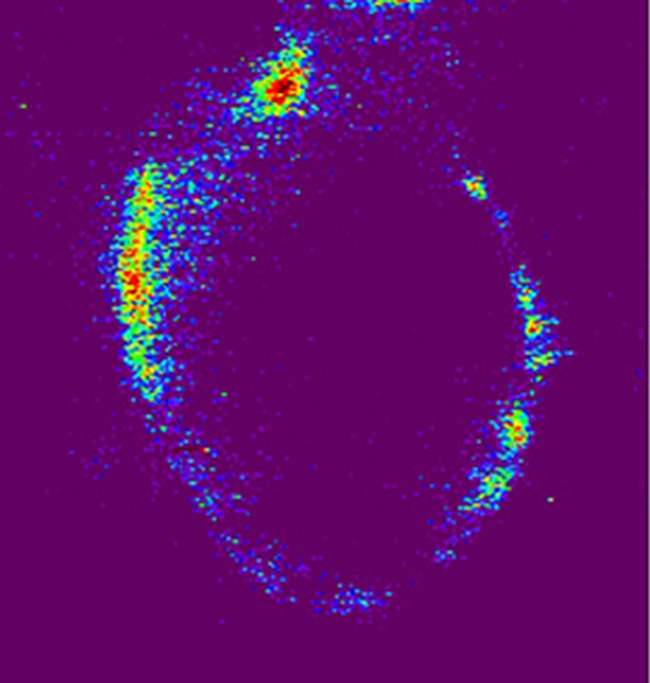

Neurons use small molecules called neurotransmitters to communicate with each other at junctions known as chemical synapses. Neurotransmitter is stored inside small sacs called synaptic vesicles, and is released into the synaptic cleft of the synapse when a vesicle fuses with the cell membrane. This process, which is known as exocytosis, can release neurotransmitter in less than a millisecond. However, it takes much longer to retrieve fused vesicle membrane to make a new vesicle (Figure 1): the fast version of this endocytosis process typically takes seconds, whereas a slow mode of endocytosis takes tens of seconds. This means that if a neuron is continuously active for a long period of time, its pool of vesicles can be depleted. Studies of vesicle recycling are complicated because the various processes involved, including membrane retrival, vesicle refilling, and transport of vesicles to the sites of exocytosis (active zones), are interdependent (Figure 1; Hosoi et al., 2009; Hua et al., 2013).

**Figure 1.**
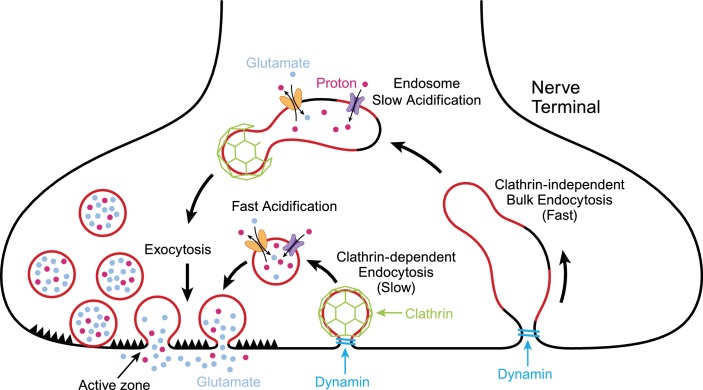
Exocytosis and endocytosis at nerve terminals. During exocytosis, synaptic vesicles (red circles) fuse with the plasma membrane at active zones (left) to release neurotransmitter molecules (glutamate; pale blue) and protons (red dots) into the synaptic cleft. Synaptic vesicles that have fused to the plasma membrane are then recycled to make new vesicles in a process involving slow (middle) or fast (right) endocytosis. Synaptic vesicles are more acidic than the cytoplasm due to the action of pump proteins (purple) that load protons into the vesicles. Transport proteins (yellow) load glutamate into vesicles in exchange for protons. Slow endocytosis (middle) relies on a protein called clathrin (green), with membrane retrieval and acidification happening at approximately the same time. Fast endocytosis involves the production of large structures called endosomes that slowly become more acidic due to the action of proton pumps. New synaptic vesicles then bud from the endosome in a process that depends on clathrin. Both modes of endocytosis require dynamin (turquoise), a protein that pinches off the membrane.

Two techniques have been widely used to study vesicle recycling at synapses: patch clamping and fluorescent imaging. The patch clamp technique can be used to measure changes in the capacitance of the cell membrane and is a direct way to track membrane endocytosis (von Gersdorff and Matthews, 1994). Fluorescent imaging involves attaching pH-sensitive dyes to proteins in the vesicle membrane and recording how the fluorescence signal from the dye changes in response to fluctuations in pH (the inside of a vesicle is much more acidic than the cytoplasm and the environment outside the cell; Fernández-Alfonso and Ryan, 2004). When neurons are moderately stimulated, these two techniques report approximately the same time course, corresponding to the slow mode of endocytosis. However, stronger stimulation leads to conflicting results: patch clamp studies suggest that a fast mode of endocytosis becomes dominant, whereas fluorescent imaging reports a slowed time course for vesicle recycling.

Now, in eLife, Mitsuhara Midorikawa at Doshisha University and co-workers – including Yuji Okamoto as first author – report an elegant series of experiments where they used both patch clamping and fluorescent imaging at the same time to investigate vesicle recycling at a nerve terminal called the calyx of Held in rodents (Okamoto et al., 2016). Following moderate stimulation of the nerve terminal, patch-clamp experiments revealed the presence of both fast and slow modes of membrane endocytosis. However, fluorescent imaging revealed a delayed and slow time course for the pH change corresponding to the slower mode of endocytosis only. Nevertheless, both techniques reveal a significant block of endocytosis when small molecules that target the function of a critical protein called dynamin are introduced into the nerve terminal (Yamashita et al., 2005; Delvendahl et al., 2016).

When a stronger and prolonged stimulus was used, the fast form of endocytosis dominated according to membrane capacitance measurements, while the fluorescent signal reported almost no recovery of the acidic pH in vesicles for about 30 seconds after exocytosis. This crucial experiment reminds us that fluorescent imaging merely reflects the process by which the new vesicles are filled with protons (or re-acidification; see Figure 1), not the retrieval of membrane itself. Re-acidification might be much slower than membrane retrieval, particularly during fast endocytosis, which may be mediated by bulk endocytosis and the formation of transient endosomes that then bud off synaptic vesicles (Figure 1; de Lange et al., 2003; Watanabe et al., 2014). Ultimately, measuring membrane capacitance appears to be more reliable than fluorescent imaging as a tool for reporting synaptic vesicle membrane retrieval. Okamoto et al. also provide evidence that inhibiting a specific calcium-sensitive signaling pathway at active zones can prevent vesicle proteins from being taken up without affecting the retrieval of membrane. However, it is not clear whether this “decoupling” plays a biological role under physiological stimulation conditions.

Previous studies have shown that calcium ions both inhibit and promote endocytosis under various conditions (Hosoi et al., 2009; Leitz and Kavalali, 2011). The results of Okamoto et al. will be useful for designing experiments to clarify the distinct roles of calcium ions in regulating the different modes of endocytosis. Their approach could also be extended to use conditions that more closely match the normal activation patterns of neurons in the brain, where vesicle recycling happens very quickly at physiological temperatures (Delvendahl et al., 2016).

The slow mode of endocytosis depends on a protein called clathrin to make vesicles from the cell membrane or from endosomes (López-Murcia et al., 2014). Recently researchers in the UK observed a new role for clathrin in coordinating vesicle recycling in a ribbon-type chemical synapse on a faster time scale than seen previously (Pelassa et al., 2014). Further investigation is required to determine if this role for clathrin is specific to ribbon-type synapses, or whether it also applies to other types of synapses. Moreover, Pelassa et al. also found that the timing of the changes in the fluorescent signal and the membrane capacitance corresponded well with each other for a single brief stimulus condition. However, Okamoto et al. have demonstrated that there is much insight to be gained from studying strongly stimulated neurons where this correspondence breaks down.
